# Comparative Analysis of Large Language Model and Physician-Generated Responses in Bariatric Patient Inquiries: Assessing the Accuracy and Patient Satisfaction

**DOI:** 10.1007/s11695-025-08115-w

**Published:** 2025-08-01

**Authors:** Katharina Vedder, Susanne Blank, Tea Wilhelm, Darick Fidan, Ihor Pachkiv, Han Cao, Christel Weiß, Chengpeng Li, Marion Rung-Friebe, Christoph Reissfelder, Mirko Otto, Cui Yang

**Affiliations:** 1 Department of Surgery, Medical Faculty Mannheim, University Medicine Mannheim, University of Heidelberg, Mannheim, Germany; 2 Clinic for General and Visceral Surgery, Bethesda Evangelical Hospital, Duisburg, Germany; 3 Central Institute of Mental Health, Mannheim, Germany; 4 Department of Medical Statistics and Biomathematics, Medical Faculty Mannheim, Heidelberg University, Mannheim, Germany; 5 Key Laboratory of Carcinogenesis and Translational Research (Ministry of Education/Bejing); Sarcoma Center, Peking University Cancer Hospital, Beijing, China; 6 Obesity Association Germany, Bottrop, Germany; 7 AI Health Innovation Cluster, German Cancer Research Center, Heidelberg, Germany

**Keywords:** Obesity, Patient education, Patient communication, Bariatric surgery, LLM, Patient-centered survey

## Abstract

**Background:**

Large language models (LLMs) can generate human-like, empathetic responses within seconds. Their potential in terms of comprehensibility, empathy, and completeness to support physician–patient communication in bariatric surgery care needs to be evaluated.

**Methods:**

We collected 200 real-world questions from patient support groups, initial consultations, and follow-up visits, which were answered by GPT-4o and two human bariatric experts. An independent bariatric expert then blindly evaluated the responses for their overall quality, accuracy, and comprehensiveness. If needed, the responses were corrected, and the correction time was documented. Afterwards, bariatric patients (*n* = 189) across Germany rated the responses, assessing each one on its clarity, empathy, and completeness.

**Results:**

The LLM required significantly less time (2.7 vs. 87.2 s, *p* < 0.0001) and generated longer responses (607 vs. 262 characters, *p* = 0.001) than human experts. LLM-generated responses were rated significantly higher by patients in terms of clarity (4.8 vs. 4.6), completeness (4.5 vs. 3.4), and empathy (4.1 vs. 3.2, all *p* < 0.0001). In total, 64.9% of patients preferred LLM-generated responses, while 18.5% preferred physician responses. Notably, patients with a lower degree of education showed a stronger preference for LLM responses over physician responses.

**Conclusion:**

LLMs could possibly act as an assistant for physicians and help improve their response efficiency while maintaining accuracy under physicians’ oversight. This approach could optimize physician time management and enhance patient satisfaction in bariatric care communication.

**Supplementary Information:**

The online version contains supplementary material available at 10.1007/s11695-025-08115-w.

## Introduction

Large language models (LLMs) are an advanced form of artificial intelligence (AI) which can understand and generate human-like text after being trained on huge amounts of data covering a broad range of topics. Generative pre-trained transformer (GPT) is an LLM which demonstrates ability in generating responses to user queries, including domain-specific topics such as healthcare. Since it was launched by OpenAI in 2022, GPT’s potential in improving patient care and medical research, [1–5] as well as its limitations, [6, 7] has been discussed by healthcare providers and researchers. By providing well-written and conversational responses, GPT has the potential to play a significant role in patient education [8] and facilitate shared decision-making [9], thereby enhancing patient understanding and engagement.

Obesity has become a significant health issue worldwide. For individuals facing severe obesity, bariatric surgery has proven to be the most effective treatment, often resulting in sustained weight loss for years postoperatively [10] and decreasing the incidence of obesity-related comorbidities, such as diabetes, hypertension, sleep apnea, and cardiovascular diseases [11–13]. Patient education and guidance are critical in the pre- and postoperative phases to ensure successful outcomes. During the process, patient inquiries usually need to be addressed by healthcare professionals effectively. Previous studies have investigated the accuracy, reproducibility, comprehensiveness, and readability of LLM-generated responses to frequently asked bariatric-relevant questions [14–17]. The percentages of comprehensive responses varied from 66.7% to 93.8%, depending on question types [16]. Goh et al. [18] analyzed if LLMs could improve the diagnostic thinking of physicians compared to conventional resources. The results showed that the LLM did not give a significant benefit to clinical decision making but was able to perform better than physicians alone, which suggests the potential of cooperation between physicians and AI [18]. Despite all their potential, LLMs harbor some known limitations that raise concerns about their application in healthcare. Unlike doctors, who utilize solid foundations in medical knowledge and years of practical experience, AI systems such as GPT-4 are based on existing data patterns and text connections. Its propensity for “hallucinations,” the phenomenon where LLMs generate seemingly correct but inaccurate, misleading information, might endanger patients’ safety if used in a completely independent implementation [19]. Moreover, as an AI tool, LLMs might not be able to provide human elements such as a nuanced understanding of individual patient circumstances and emotional support.

In this study, we hypothesized that LLMs could be used as an assistant by drafting initial responses to patient inquiries. We compared response generation times, time required for correction, and patient satisfaction between LLM-assisted and traditional physician responses to assess whether this hybrid model could enhance both healthcare provider efficiency and patient satisfaction while maintaining medical accuracy.

## Methods

### Study Design

We conducted a prospective, single-blind, randomized study comparing responses provided by GPT-4o and human bariatric experts. The study was approved by the local Ethical Committee and conducted at a university hospital from January to May 2024. It was registered in the German Clinical Trials Register. Informed consent was implied by survey response. The trial was overseen by a representative from patient support groups.

**Fig. 1:**
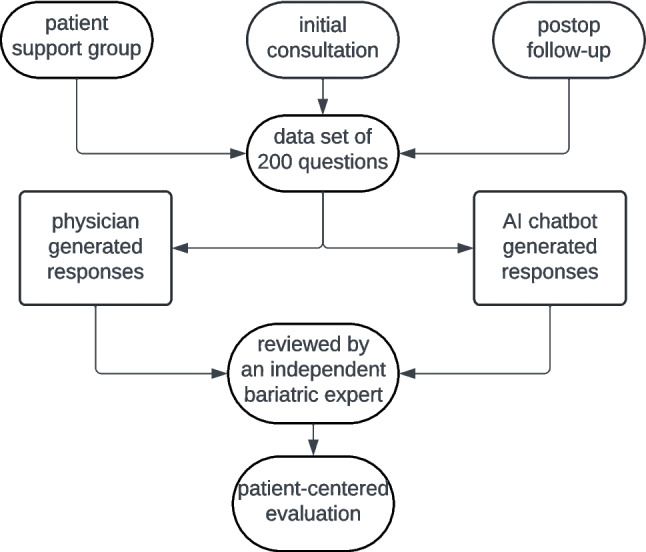
Flowchart of the trial

The trial workflow is presented in Fig. 1.

### Question Collection

Real-world patient queries were collected from diverse sources to represent a wide range of concerns regarding bariatric surgery: patient support groups, initial consultation at our bariatric center, and follow-up visits after bariatric surgery (both in-person and mobile app-based). The distribution of questions from different sources was based on availability during our collection period. To reflect the real-world situation, we documented all questions asked by patients. Only duplicate questions were excluded from the dataset. When uncertainty appeared regarding question inclusion, the corresponding author made the final decision on whether the question should be included in the study. Questions were not reworded and were kept in their original form to maintain authenticity of patient inquiries. In this phase, particular attention was paid to protect patient privacy by ensuring that no personally identifiable information was collected. A dataset of 200 questions was generated.

### Generation of Responses by Human Experts and the LLM

All collected questions were presented in German and answered separately by human bariatric experts (coauthors MO and SB) and GPT-4o in German.

Human experts were required to answer the questions and document the time needed. Additionally, human experts needed to rate the difficulty level (easy, medium, and hard). Examples of different levels included:

- Easy: “For how long do I have to pay particular attention to my protein intake after bariatric surgery?”
- Medium: “How can I avoid late dumping after my bariatric surgery and what can I do acutely if I have it?”
- Hard: “I had bariatric surgery 7 years ago and another one a year ago (distalization of gastric bypass) due to weight gain. Since the second operation I have lost 20 kg from 133 kg quite quickly, but hardly ever since. Why is that? What can I do?”

For example, questions and answers are shown in Table 1.

**Table 1:** Examples of LLM and human expert generated answers to patient questions, translated from German

Question source	Difficulty level	Human expert-generated response	LLM-generated response
How much should I drink every day after bariatric surgery?	Easy	As for all people, the recommendation is 2–3 L, but this is often difficult in the first 6 months. Then at least 1 L	After bariatric surgery, you should drink around 1.5 to 2 L a day. It is important that you drink small sips throughout the day and avoid carbonated drinks
Is it okay to take cefuroxime for otitis media after bariatric surgery?	Medium	Cefuroxime can also be taken after bariatric surgery. In principle, bypass operations can lead to a reduced absorption of medication. However, it can be assumed that cefuroxime is sufficiently effective	Yes, taking cefuroxime is usually fine after bariatric surgery as long as there are no specific contraindications or allergies. It is important that you take the medication according to the instructions and watch out for possible side effects. If you notice any unusual symptoms, please let me know
Can I fast on Ramadan with my gastric bypass?	Hard	The problem with fasting after a bypass is that it is not possible to consume enough food for the whole day in a short space of time, as the portion you can eat at once is smaller. There is no general ban on fasting, but great care must be taken to ensure that sufficient fluids and nutrients are consumed. For this reason, we do not recommend fasting after gastric bypass	After gastric bypass, fasting during Ramadan is complex. It is important to carefully monitor your food and fluid intake to avoid complications. You should eat small, frequent meals and drink plenty of fluids, which is made more difficult by fasting. I recommend that you avoid fasting or work with a nutritionist and myself to create a personalized plan that considers your health needs

For all questions and answers generated by human experts and the LLM, which were translated into English with the help of DeepL (https://www.deepl.com/de/translator), please see supplementary table S1

For LLM-generated responses, we interacted with GPT-4o through the OpenAI Application Programming Interface (API) (https://openai.com/blog/openai-api; accessed on 14 May 2024). We used the following settings of GPT-4o: temperature = 0.7 (parameter of LLM that controls the randomness and creativity in the model’s responses), context window = 128,000 (maximum tokens allowed per input), max output tokens = 16,384 (longest allowed length of generated responses). API calls were made using Python with the OpenAI library. Response generation time was measured from the moment the API request was initiated until the complete response was received. All API calls were made from the same institutional network connection to ensure consistent conditions. Time measurements were automatically recorded using Python’s time module. Each question was processed individually through separate API calls to accurately measure per-question response times.

We provided GPT-4o with the following specific prompts to prime the model before any generation: “Act as a bariatric expert, ensure that your responses are as helpful and safe as possible, and avoid providing any potentially harmful advice or information. If you don’t know the answer, just answer “I don’t know” instead of making up facts.” By assigning the role of a bariatric expert, we aimed at guiding the LLM with a specific role and background to sharpen the relevance of its responses. Phrases indicating that the responses were generated by a LLM such as “Please note that I am an AI developed by OpenAI, designed to provide information and suggestions based on general knowledge and data.” were deleted prior to further evaluation.

### Quality Assessment by an Independent Expert

An independent third-party bariatric expert (coauthor FD), who was blinded to the source of the responses (LLM or human bariatric experts), rated the responses based on overall quality, accuracy, and comprehensiveness. If necessary, FD corrected the responses and documented the time needed. To correct responses generated by LLM, ChatGPT-4o (https://chatgpt.com) was utilized to assist with revision, with specific errors or omissions highlighted for correction. The prompts were also written in German. Here is an example of an answer from LLM that had to be corrected:

- Question: Is gastric banding an alternative to gastric bypass or gastric sleeve?‬
- Answer GPT-4o: Yes, the gastric band is an alternative to gastric bypass and sleeve surgery. It is less invasive and can be adjusted or removed. However, it usually has lower weight loss results and can lead to complications such as band erosion or displacement.‬
- Corrector: However, the gastric band is rarely used anymore because of the complications and poorer results, so it is not an equivalent alternative. Can you rephrase the answer to take this into account?‬
- GPT-4o: You are right. Here is a revised version: While gastric banding was considered a less invasive alternative to gastric bypass and gastric sleeve in the past, it has been shown to be less effective due to frequent complications such as band erosion or displacement and lower weight loss results. For these reasons, gastric banding is rarely used today and is not an equivalent alternative to gastric bypass or sleeve gastrectomy. Instead, many bariatric surgeons are focusing on procedures that have higher success rates and lower complication rates.‬

### Patient Evaluation Using an Online Survey

The 200 questions were randomly divided into 20 subsets, each consisting of 10 questions and their corresponding responses generated by LLM and by human bariatric experts. These were further compiled into an anonymous online survey, where participants were provided with a brief explanation of the study’s purpose and asked for consent to collect minimal demographic information, including age, gender, and education level.

To minimize bias, participants were randomly assigned one subset and were blinded to the source of these responses. The appropriateness of length, clarity, completeness, and satisfaction as well as empathy were rated using a 5-point Likert scale. Except for length, 5 was the best possible score and 1 was the worst. Additionally, they chose the preferable answer to the questions.

### Statistical Analysis

The patient-based ratings were analyzed to evaluate differences in patient satisfaction and perceived empathy between the AI-generated and human expert-provided answers. Time needed for correction was also compared. For statistical analysis, SAS software, release 9.4 (SAS Institute, Cary, NC, USA) was used. Quantitative variables are presented as the mean ± standard deviation; for qualitative factors, absolute and relative frequencies are given. Wilcoxon tests for two paired samples were used to compare continuous and ordinally scaled variables between LLM and physician responses. These comparisons have been performed separately according to the difficulty levels (easy, medium, and hard). To compare the ratings of LLM responses between two independent samples (i.e., male/female participants), the two-sample Wilcoxon test has been used. For factors with more than two categories (i.e., education), the Kruskal–Wallis test has been used instead. In general, the result of a statistical test was considered significant when *p* < 0.05 (*).

## Results

### Question Source, Type, and Difficulty Level

Between January and May 2024, we collected 200 real-world questions related to bariatric surgery, among which 41 (20.5%) derived from patient support groups, 159 (79.5%) from initial consultation at the bariatric center and from follow-up visits after bariatric surgery. The bariatric experts categorized 97 questions (48.5%) as easy, 90 (45.0%) as medium, and 13 (6.5%) as hard based on medical complexity.

### Generation of Quality of Responses

For all questions, human bariatric experts needed significantly more time to generate responses (219.1 vs. 8.7 min total). Time needed to generate responses varied by question difficulty: human experts needed 54.2 ± 34.9 s for easy, 81.5 ± 47.8 s for medium, and 125.9 ± 81.3 s for hard questions. In comparison, the LLM generated answers in 2.4 ± 1.6 s, 2.7 ± 1.3 s, and 3.0 ± 2.3 s, respectively (*p* < 0.0001 for easy and medium questions, *p* = 0.0002 for hard questions).

Human-generated responses were significantly shorter than LLM-generated answers measured by the number of characters (262.6 vs. 607.5, *p* = 0.001). This difference is present through all categories (easy: 161.7 ± 108.9 vs. 489.4 ± 278.6, *p* < 0.0001; medium: 281.6 ± 123.6 vs. 674.2 ± 369.9, *p* < 0.0001; hard: 344.5 ± SD 190.4 vs. 658.9 ± 335.6, *p* = 0.001 respectively).

Overall quality was rated on a 5-point Likert scale (1–5). For easy questions, LLM answers achieved the best score (5) more frequently than physician answers for both easy (89.7% vs. 61.9%, *p* < 0.0001) and medium questions (83.3% vs. 61.1%, *p* = 0.004). There were no significant differences in response quality for hard questions. However, both LLM and physician responses showed similar accuracy and completeness across all difficulty categories.

### Correction of Responses

Based on the independent bariatric expert’s assessment, 13 (6.5%) of the LLM-generated responses required correction and 9 (4.5%) of the human expert-generated responses required revision.

Among the LLM-generated responses requiring correction, one of the answers was categorized as potentially dangerous for patients because critical information was missing (supplementary table S2). For physician-generated responses requiring corrections, 77.8% of corrections took less than 1 min and 22.2% 1–3 min. For LLM-generated responses needing correction, 15.2% required under 1 min and 84.6% 1–3 min.

### Patient-Centered Evaluation

#### Participants’ Demographics

Of 189 participants who responded to the survey, 165 (87.3%) were females, with a mean age of 48.2 ± 11.2 years (range, 21–76). Regarding the self-reported highest educational qualification, 163 (86.2%) had secondary qualifications (19.6% lower, 48.7% intermediate, 18% college entrance qualification), 23 (12.2%) university degree (bachelor, master, doctorate), and 3 (1.6%) no formal qualification.

#### Response Quality Assessment

The best possible rating for the answer length was “3” (“1” was considered far too short, “5” was considered far too long). Regardless of the level of difficulty, LLM responses outperformed physician responses across all aspects: LLM responses were rated closer to the optimal length (3.4 vs. 2.4; *p* < 0.0001), clearer (4.8 vs. 4.6; *p* < 0.0001), more complete (4.5 vs. 3.4; *p* < 0.0001), and more empathetic (4.1 vs. 3.2; *p* < 0.0001) (Table 2).

**Table 2:** Patients’ rating of the length, clarity, completeness, and empathy of LLM and humans’ responses for all answers

	Physician generated	LLM generated	Difference	*p*-values
Appropriateness of length—all answers	2.4	3.4	1	** < 0.0001**
Easy (*N* = 97)	2.3	3.3		
Medium (*N* = 90)	2.5	3.4		
Hard (*N* = 13)	2.6	3.5		
Clarity—all answers	4.6	4.8	0.2	** < 0.0001**
Easy (*N* = 97)	4.6	4.8		
Medium (*N* = 90)	4.6	4.8		
Hard (*N* = 13)	4.6	4.7		
Completeness and satisfaction—all answers	3.4	4.5	1.1	** < 0.0001**
Easy (*N* = 97)	3.3	4.5		
Medium (*N* = 90)	3.5	4.6		
Hard (*N* = 13)	3.6	4.6		
Empathy—all answers	3.2	4.1	0.9	** < 0.0001**
Easy (*N* = 97)	3.0	4.0		
Medium (*N* = 90)	3.3	4.1		
Hard (*N* = 13)	3.3	4.1		

Significant *p*-values are highlighted in bold. Means are presented for quantitative data

The quality of physician responses improved when the question difficulty increased: the ratings were higher for empathy (3.3 vs. 3.0, *p* =  < 0.0001), length (2.6 vs. 2.3, *p* < 0.0001), and completeness (3.6 vs. 3.3, *p* = 0.0003) for medium/hard questions when compared to easy ones. For LLM responses, there were no significant differences between the difficulty levels for the ratings (Table 3).

**Table 3:** Patients’ ratings of the length, clarity, completeness, and empathy by the difficulty level of the questions

	Responder	Easy	Medium	Hard	*p*-value
Empathy	Physicians	3	3.3	3.3	** < 0.0001**
	LLM	4	4.1	4.1	0.8
Length	Physicians	2.3	2.5	2.6	** < 0.0001**
	LLM	3.3	3.4	3.5	0.08
Clarity	Physicians	4.6	4.6	4.6	0.7
	LLM	4.8	4.8	4.7	0.06
Completeness	Physicians	3.3	3.5	3.6	**0.0003**
	LLM	4.5	4.6	4.6	0.4

Significant *p*-values are highlighted in bold. Means are presented for quantitative data

#### Demographic Influences

Gender significantly affected response preferences. Women gave higher ratings to LLM responses for empathy (4.1 vs. 3.9, *p* = 0.008), clarity (4.8 vs. 4.7, *p* = 0.0003), and completeness (4.6 vs. 4.4, *p* = 0.02). Men rated physician responses significantly higher for empathy (3.3 vs. 3.1, *p* = 0.02), length (2.6 vs. 2.3, *p* = 0.003), and completeness (3.6 vs. 3.4, *p* = 0.004).

Educational background also influenced preferences. Lower-educated participants rated physician responses higher for empathy, length, and completeness, while higher-educated participants gave better ratings to LLM responses for completeness (Table 4).

**Table 4:** Patient ratings of the length, clarity, completeness, and empathy by gender and educational level

	Responder	Male	Female	*p* Gender	No form. qual	LSQ	ISQ	SREQ	CEQ	University degree	*p* Education
Empathy	Physicians	3.3	3.1	0.02	4.3	3.4	3.2	2.8	2.8	3.3	< 0.0001
	LLM	3.9	4.1	0.008	4.2	4.1	4	4	3.9	4.1	0.002
Length	Physicians	2.6	2.3	0.003	3.8	2.5	2.3	2.1	2.1	2.5	< 0.0001
	LLM	3.4	3.4	0.2	3.8	3.4	3.4	3.3	3.3	3.4	0.09
Clarity	Physicians	3.3	4.6	0.3	4.7	4.6	4.5	4.7	4.8	4.8	< 0.0001
	LLM	4.7	4.8	0.0003	4.7	4.8	4.8	4.8	4.8	4.9	0.03
Completeness	Physicians	3.6	3.4	0.004	4.3	3.5	3.4	3.2	3.3	3.7	< 0.0001
	LLM	4.4	4.6	0.02	4.3	4.4	4.6	4.5	4.6	4.6	0.0002

*LSQ*, lower secondary qualifications; *ISQ*, intermediate secondary qualifications; *SREQ*, subject-related entrance qualification; *CEQ*, college entrance qualification

Overall, 64.9% of participants preferred LLM-generated responses, 18.5% favored physician responses, and 16.6% rated both equally.

## Discussion

Our study demonstrates that GPT-4o, when supervised by human experts, could generate responses which were rated significantly better by participants in terms of empathy, clarity, and completeness, with approximately 65% of participants preferring LLM-generated answers than those generated by physicians without LLM involvement. Gender and educational background seem to have an impact on ratings and preferences.

Our findings are in line with previous research examining AI performance in the medical field. In a study of Kim et al. analyzing patient medical advice requests in electronic health records (EHRs), AI-generated responses were rated significantly higher in satisfaction compared to clinicians’ responses, especially for cardiology and endocrinology questions [20]. Samaan et al. analyzed the accuracy of ChatGPT-3.5’s responses to bariatric surgery questions and showed that ChatGPT could provide comprehensive answers (best score) in 87% of all questions, with particularly strong performance (94%) in questions on “Effectiveness, suitability and treatment options.” [21] Similarly, Leng et al. evaluated the responses generated by ChatGPT-4 in answering 30 questions about bariatric surgery. Fifty percent of the answers were in line with current guidelines/expert consensus and received the highest rating. Forty-seven percent of the responses were intermediate and only one response (3%) was inadequate [22].

Even though the accuracy rates were high to very high, LLMs alone still made mistakes which could cause harm to patients. The findings of these previous trials emphasize the need for supervision by human experts, as performed in our trial. Moreover, these trials have focused on the accuracy evaluated by clinical experts without considering the patient perception. Previous studies have shown the feasibility of using LLMs to improve health literacy. Srinivasan et al. demonstrated the efficacy of LLMs in enhancing the readability of bariatric surgery patient education materials [14]. A review demonstrated the potential of LLMs in improving doctor-patient communication in various aspects [23]. Our patient-centered survey confirmed the advantages that LLMs could offer perioperative care instructions and optimize interactions using patient-friendly language.

We chose to utilize GPT-4o instead of previous models due to several reasons. Firstly, compared to the earlier versions, GPT-4o should be able to address more complex problems with greater precision due to the more advanced algorithms and the significantly larger training dataset [24]. It demonstrated significantly better performance than ChatGPT-3.5 in answering questions in different medical fields [25–29] and also has been specifically improved to reduce hallucination problems (generation of plausible but factually incorrect or misleading information) [30].

We were aware that the performance of AI LLMs is significantly influenced by the form and choice of wording of the entered queries, usually referred to as “prompts.” [31] To leverage the capabilities of the AI model and limit any potential biases that could affect the outcomes, we standardized our prompts. By assigning the AI the role of a bariatric expert, we aimed to enhance the relevance and accuracy of its responses. However, it is important to note that for queries specifically related to nutrition, a bariatric expert may not be the ideal source of information compared to a nutrition specialist. Nonetheless, given our study’s focus on the feasibility of LLMs as an assistant by drafting initial responses to patient inquiries to save time for bariatric experts, we maintained the designated role of bariatric expert throughout the investigation.

Our results suggest a promising hybrid approach in which LLMs write initial responses that are reviewed and revised by physicians before transmission to patients. This approach could significantly improve efficacy while maintaining patient safety and quality standards. The standardization of prompts provides a framework of consistent, high-quality responses. However, adaptation might be necessary where subspecialty expertise (such as nutrition) is needed.

Our study has several limitations. First, the answers were generated by two bariatric experts, and our evaluation relied on one single bariatric expert as a corrector, potentially limiting the generalizability of our physician-generated response comparisons. Second, 87% of our participants were female, which may affect the generalizability of the patient satisfaction findings. Third, our conclusions are based on ChatGPT-4o, and more advanced models such as OpenAI o1 or DeepSeek-R1 might show different performance. Fourth, the response quality could potentially vary in different languages. We have only analyzed the performance in German. Fifth, the temperature was set to 0.7, balancing clinical accuracy with conversational naturalness. Using a lower temperature setting might increase the reproductivity of the answers. It may be presumed that the reproducibility of answers between different doctors increases with professional experience. With a highly trained AI that has been set to a lower temperature, it is presumably possible to achieve a higher level of answer reproducibility than if different physicians with different levels of experience answer the same question.

## Conclusion

LLMs demonstrate significant potential as copilots in bariatric surgery patient communication, offering responses with enhanced empathy and clarity while maintaining clinical accuracy. While LLMs still have the risk of providing misinformation, this can be effectively mitigated through human expert oversight. These findings contribute to the growing body of evidence supporting the carefully supervised integration of AI technologies into the healthcare section and highlight the importance of maintaining human oversight in medical decision-making and patient care.

## Supplementary Information

Below is the link to the electronic supplementary material.

Supplementary file1 (XLSX 75 KB)

Supplementary file2 (DOCX 25 KB)

## Data Availability

No datasets were generated or analysed during the current study.
