# Supplementary material for: Comparative Analysis of Large Language Model and Physician-Generated Responses in Bariatric Patient Inquiries: Assessing the Accuracy and Patient Satisfaction
Source: Obes Surg. 2025 Aug 1;35(9):3801–9. doi: 10.1007/s11695-025-08115-w (PMC12457575; doi:10.1007/s11695-025-08115-w)
Supplement: Supplementary file 2 — Supplementary file2 (DOCX 25 KB) [file 11695_2025_8115_MOESM2_ESM.docx]

**Table S2** Questions with original answers from Chat GPT and the corrected version by ChatGPT.

| Question | Answer | Corrected answer | Corrected content | Rating by bariatric expert |
| --- | --- | --- | --- | --- |
| I had bariatric surgery 2 months ago. Now I have massive cramps in my lower abdomen, a fever of 39°C and diarrhoea. I have the feeling that when I drink water, it runs straight through. Where does this come from and what can I do? | I am sorry to hear that you are not feeling well. Your symptoms could indicate a post-operative complication such as infection, dehydration or anastomotic insufficiency. The fever and diarrhoea are particularly worrying. It is important that you come to our clinic or visit the emergency department immediately so that we can carry out a thorough examination and determine the cause of your symptoms. In the meantime, you should try to drink small amounts of clear fluids to prevent dehydration and rest. Please do not hesitate to seek medical attention immediately. | I am sorry to hear that you are not feeling well. Your symptoms could indicate a post-operative complication such as infection, dehydration or anastomotic insufficiency. Although there are less serious causes such as a gastrointestinal infection that could explain your symptoms, the fever and diarrhoea are particularly worrying and should be investigated quickly. It is important to come to our clinic or visit the emergency department immediately so that we can carry out a thorough examination and determine the cause of your symptoms. In the meantime, you should try to drink small amounts of clear fluids to prevent dehydration and rest. Please do not hesitate to seek medical attention immediately. | Added information: There are also harmless reasons that have nothing to do with the surgery. | No dangerous misinformation, the correction is more detailed. |
| When can I start taking metformin after bariatric surgery? | Usually you can start taking Metformin again as soon as you are able to consume liquids and food without problems and your gastrointestinal tract is stabilised. This is usually the case about 1 to 2 weeks after the operation. I will monitor your individual recovery and let you know the exact time frame. | As a rule, metformin should initially be paused after bariatric surgery. After surgery, blood sugar often stabilises quickly and it is important that antidiabetic medication is readjusted. In addition, there is a risk of metabolic acidosis, which could be exacerbated by metformin. Your treating physician will monitor your blood glucose levels closely and tell you when and if you can resume metformin based on your individual recovery and stabilisation of blood glucose. It is important to work closely with your medical team during this time. | The indication for metformin after bariatric surgery should be checked again; it may no longer be necessary. Note on the risk of metabolic acidosis. | Not a dangerous answer, but first answer in general about surgery and not specifically about bariatric surgery. |
| How much will I have to pay after bariatric surgery? | The costs after bariatric surgery can vary, but they usually include post-operative care, nutritional counselling, possible medication and regular check-ups. It is also important to consider possible complications that may incur additional costs. We will discuss these factors individually to give you a more accurate estimate. Please let us discuss your exact insurance situation and specific requirements so that we can clarify the exact costs. | After bariatric surgery, you may incur various costs, which usually include post-operative care, nutritional counselling and regular check-ups. In Germany, however, the costs of necessary medication, follow-up examinations and the treatment of complications are generally covered by health insurance, provided that the operation has been authorised by the health insurance company.  It is nevertheless advisable to find out in advance from your health insurance company exactly which specific services are covered and whether there may be additional costs not covered by the insurance, for example for special nutritional programmes or additional support from specialists. By discussing your individual insurance situation and specific requirements, we can give you a more accurate estimate of the possible costs. | If bariatric surgery is correctly indicated, most costs such as surgery, consultations, nutritional counselling etc. are covered by health insurance in Germany. | Answer not correct because German healthcare system is not considered. No medical misinformation. |
| I had bariatric surgery 12 weeks ago. I've had a headache for a few days now. Could these be deficiency symptoms? Would a blood test help? | Yes, headaches could indicate deficiency symptoms, especially if you are not taking enough vitamins and minerals after the operation. A blood sample would be useful to check your values and identify possible deficiencies. This will allow us to take targeted corrective treatment. Please make an appointment for a blood test. | Headaches after bariatric surgery could be caused by various factors and may not necessarily indicate deficiency symptoms. It is certainly important to keep an eye on your wellbeing. While it is true that deficiencies could cause headaches after bariatric surgery, this is quite rare. Initially, you can use suitable painkillers and observe whether the headaches subside. If the headaches persist or other symptoms occur, it would be advisable to have a blood sample taken to ensure that there are no nutritional deficiencies. In this case, please make an appointment with your doctor to allow a detailed examination. | Added information: There are also reasons for headaches that are separate from the operation and are not due to deficiency symptoms. | Correction response much more accurate and correct. But no dangerous misinformation. |
| I had bariatric surgery 3 weeks ago. I ate a whole watermelon yesterday and then had trouble sleeping the same evening. When I measured my blood sugar, it was 200 mg/dl. Could the sleep problems have been due to the high blood sugar? | Yes, that is possible. High blood sugar can lead to sleep problems. It would be advisable to limit the amount of fruit you eat in future, especially sugary fruit such as watermelon. Make sure you eat a balanced diet and measure your blood sugar regularly. If the problems persist, we should consider further tests. | It is unlikely that the watermelon itself was the problem. Nevertheless, high blood sugar can certainly lead to sleep problems, especially after bariatric surgery, as the body is still sensitive to food changes. A blood sugar level of 200 mg/dl is elevated and could lead to discomfort. It would be advisable to monitor and moderate the amount of fruit, especially sugary fruit such as watermelon, in the future. Make sure you eat a balanced diet and measure your blood sugar regularly. If the sleep problems persist or other symptoms occur, it is important that you discuss this with your doctor to clarify possible causes and consider appropriate treatment. | Added information: High blood sugar can lead to sleep problems, but it is unlikely that the watermelon was the problem. | Corrected answer is a better answer, but not dangerous misinformation |
| I have to keep an exercise diary before my bariatric operation as part of the multimodal concept. Do I also have to do this if I am unable to fulfil the required amount of exercise due to pre-existing physical illnesses? | Yes, the exercise diary is still important. It helps us to get an accurate picture of your physical activity and your limitations. You should also document your previous illnesses and how they affect your ability to move. This enables us to provide the best possible care and make adjustments to meet your individual needs. | Yes, the exercise diary is still important. It helps us to get an accurate picture of your physical activity and your limitations. You should also document your previous illnesses and how they affect your ability to move. It is also important that you obtain a certificate from your orthopaedist or traumatologist. This certificate serves as proof for your health insurance company and explains why you may not be able to achieve the prescribed amount of sporting activities. In this way, we can work together to ensure that your individual situation is taken into account when planning and implementing the multimodal concept. | Added information: The exercise diary as part of the multimodal concept can be left out if there is a certificate from the orthopaedist, which the health insurance company then also requires. | Corrected answer is more accurate but not a dangerous misinformation. |
| Can bariatric surgery and the subsequent weight loss improve or completely eliminate my sleep apnoea? | Yes, losing weight after bariatric surgery can significantly improve or even eliminate your sleep apnoea. Weight loss often leads to a reduction in fatty tissue in the throat, which clears the airways and thus reduces sleep apnoea. | Yes, losing weight after bariatric surgery can significantly improve or even completely eliminate your sleep apnoea. The main reason for this is not only the reduction of fatty tissue in the throat, but in particular the improvement in breathing mechanics. The weight loss leads to a reduction in visceral fat and a strengthening of the auxiliary respiratory muscles. These changes enable more efficient breathing and can therefore significantly reduce or eliminate the symptoms of sleep apnoea. | More correct explanation: Weight loss leads to a reduction in visceral fat which leads to better respiratory mechanics. | No dangerous misinformation. |
| I find it very difficult to drink enough after my bariatric operation (sometimes only 0.5 litres a day). What options are there for increasing the intake? | It is important that you drink enough fluids to avoid dehydration. Here are some specific tips that can help you:  1. **Small sips**: Drink small sips frequently throughout the day rather than large amounts all at once.  2 **Set reminders**: Set alarms or use apps to remind yourself to drink regularly.  3 **Vary the flavour**: Try flavouring your water with lemon slices, cucumber slices or mint leaves to make it tastier.  4 **Always have a water bottle with you**: Always have a small water bottle handy, whether you're at home or out and about.  5 **Keep a drinking log**: Keep a written record of how much you drink to develop a better awareness of your drinking habits.  6 **Liquid-rich foods**: Also take advantage of foods with high water content such as watermelon, cucumber or yoghurt.  Please try to implement these tips and let me know at our next appointment whether your drinking has improved. | [...] If you follow these tips and still find it difficult to increase the amount you drink, please seek medical advice immediately. It is important that your health is monitored regularly, especially after bariatric surgery. [...] | If the drinking volume is permanently reduced to <1 litre, a medical consultation and further diagnosis and, if necessary, intravenous volume supplementation are essential to avoid exsiccosis. | Important information is missing. Correction definitely necessary. |
| I had bariatric surgery 3 years ago and have had problems with early dumping ever since. I have now been told that Acarabose could help. Is that true? | Yes, acarbose can actually help control early dumping. It slows down the digestion of carbohydrates in the small intestine, causing blood sugar levels to rise more slowly, which can reduce the symptoms of early dumping. We can discuss the exact dosage and possible side effects to ensure this is a suitable option for you. | Yes, acarbose is typically used to treat late dumping symptoms because it slows down the digestion of carbohydrates in the small intestine and thus controls the rise in blood glucose levels. Late dumping usually occurs one to three hours after eating and is associated with a rapid rise and fall in blood glucose levels.  Early dumping, on the other hand, which occurs within 30 minutes of eating, involves too rapid emptying of stomach contents into the small intestine, often resulting in an overload of fluid and rapid distension of the small intestine.  To treat early dumping, other approaches may be helpful, such as:  1. **Smaller, more frequent meals**: Eating several small meals throughout the day can help reduce the strain on the stomach.  2 **Slow eating**: eating slowly and chewing thoroughly can slow the rate of gastric emptying.  3 **Avoidance of certain foods**: Foods rich in simple carbohydrates and sugars can make symptoms worse.  4 **Drinking between meals**: instead of drinking while eating, drinking fluids between meals can help reduce symptoms.  It is important to speak further with your treating physician or a specialised nutritionist to address your symptoms individually and find the best strategies for you. If you are still considering acarbose or would like to eliminate confusion, please discuss this in detail with your physician to ensure it is the right treatment option for your specific needs | Acarbose more for therapy of late dumping. Terms seems to have been mixed up. | Answer before correction not correct, but not dangerous. |
| Since my bariatric surgery, I always feel nauseous after eating white bread, pasta or pizza and I often vomit. Could I have developed a gluten intolerance as a result of the operation? | It is unlikely that the operation itself has caused gluten intolerance. However, it is possible that certain foods are more difficult to digest after surgery and can cause symptoms such as nausea and vomiting. White bread, pasta and pizza are often difficult to digest and can cause problems, especially if they are eaten quickly or not chewed well. I recommend avoiding these foods at first and chewing smaller portions well. We can also arrange an appointment to carry out further tests and rule out other possible causes. | It is unlikely that the operation itself has caused gluten intolerance. However, it is possible that certain foods are more difficult to digest after surgery and can cause symptoms such as nausea and vomiting. White bread, pasta and pizza are often difficult to digest and can cause problems, especially if they are eaten quickly or not chewed well.  In addition, your symptoms could indicate dumping syndrome, which occurs in some patients after bariatric surgery. In dumping syndrome, food passes too quickly from the stomach to the small intestine, which can lead to nausea, vomiting, diarrhoea, weakness and dizziness. Foods high in sugar or fat are common triggers.  I recommend avoiding these foods at first and chewing smaller portions well. Also choose foods that are high in fibre, low in fat and low in sugar. It would also be advisable to eat smaller meals throughout the day and to drink liquids between meals rather than during them.  Please make an appointment so that we can carry out further tests and rule out other possible causes for your symptoms. A detailed examination and possibly nutritional counselling could be helpful. | Added information: Note on possible dumping as a more likely reason. | Not dangerous, but first answer medically incorrect. |
| Since my gastric bypass operation I have had a lot of pimples and spots around my shoulder blades. Could this be related to the operation? Is there anything I can do about it? | Yes, it is possible that the changes in your hormonal balance and metabolism after gastric bypass surgery can lead to skin blemishes. To improve this, I recommend that you:  1. make sure you eat a balanced diet to avoid nutritional deficiencies.  2. drink enough water to keep your skin hydrated  3. use mild, non-comedogenic skincare products.  4. shower after exercise or sweating to keep pores clean.  If problems persist or worsen, we may need to consult a dermatologist. | Yes, it is possible that the changes in your hormonal balance and metabolism after gastric bypass surgery can lead to skin blemishes. To improve this, I recommend that you:  1. make sure you eat a balanced diet to avoid nutritional deficiencies. A lack of zinc in particular can lead to skin problems, so zinc supplementation may be useful. Talk to your doctor about this.  2. drink plenty of water to keep your skin hydrated.  3. use mild, non-comedogenic skin care products.  4. shower after exercise or sweating to keep pores clean.  If problems persist or worsen, we may need to consult a dermatologist. The dermatologist can not only evaluate your skin condition, but also make specific recommendations for treatment. | Added information: Possible zinc deficiency as a reason. | No dangerous misinformation. |
| I have always had rather low blood pressure. Since I had a gastric bypass 2 years ago and lost a lot of weight  and lost a lot of weight, I regularly have real problems with my circulation. The lowest pressure I measured myself was 80/60 mmHg.  I've already been to the cardiologist, and from his point of view everything is fine. What can I do to normalise this a little? | It would be useful to carry out blood sugar measurements to rule out the possibility of low blood sugar levels in the sense of a dumping syndrome. However, low blood pressure after significant weight loss is relatively common. Drinking caffeine in the morning, for example, can be helpful. The question of whether symptoms occur at the same time is also relevant. | It would make sense to see an endocrinologist to rule out the possibility of low blood sugar levels in the sense of a dumping syndrome. However, low blood pressure after significant weight loss is relatively common. Caffeine in the morning, for example, can be helpful. The question of whether symptoms occur at the same time is also relevant. |  |  |
| Is gastric banding an alternative to gastric bypass or gastric sleeve? | Yes, the gastric band is an alternative to gastric bypass and sleeve gastrectomy. It is less invasive and can be adjusted or removed. However, it usually has lower weight loss results and can lead to complications such as band erosion or displacement. | While gastric banding was considered a less invasive alternative to gastric bypass and gastric sleeve in the past, it has been shown to be less effective due to frequent complications such as band erosion or displacement and lower weight loss results. For these reasons, gastric banding is rarely used today and is not an equivalent alternative to gastric bypass or sleeve gastrectomy. Instead, many bariatric surgeons are focussing on procedures that have higher success rates and lower complication rates. | Not a recommended alternative due to the complications and poor long-term results. | First answer not dangerous but incorrect. |
